# Innovative *in Silico* Approaches for Characterization of Genes and Proteins

**DOI:** 10.3389/fgene.2022.865182

**Published:** 2022-05-18

**Authors:** Gh. Rasool Bhat, Itty Sethi, Bilal Rah, Rakesh Kumar, Dil Afroze

**Affiliations:** ^1^ Advanced Centre for Human Genetics, Sher-I- Kashmir Institute of Medical Sciences, Soura, India; ^2^ Institute of Human Genetics, University of Jammu, Jammu, India; ^3^ School of Biotechnology, Shri Mata Vaishno Devi University, Katra, India

**Keywords:** Single nucleotide polymorphisms (SNPs), Human Splice finder (HSF), Next Generation Sequencing (NGS), *in silico*, bioinformatics

## Abstract

Bioinformatics is an amalgamation of biology, mathematics and computer science. It is a science which gathers the information from biology in terms of molecules and applies the informatic techniques to the gathered information for understanding and organizing the data in a useful manner. With the help of bioinformatics, the experimental data generated is stored in several databases available online like nucleotide database, protein databases, GENBANK and others. The data stored in these databases is used as reference for experimental evaluation and validation. Till now several online tools have been developed to analyze the genomic, transcriptomic, proteomics, epigenomics and metabolomics data. Some of them include Human Splicing Finder (HSF), Exonic Splicing Enhancer Mutation taster, and others. A number of SNPs are observed in the non-coding, intronic regions and play a role in the regulation of genes, which may or may not directly impose an effect on the protein expression. Many mutations are thought to influence the splicing mechanism by affecting the existing splice sites or creating a new sites. To predict the effect of mutation (SNP) on splicing mechanism/signal, HSF was developed. Thus, the tool is helpful in predicting the effect of mutations on splicing signals and can provide data even for better understanding of the intronic mutations that can be further validated experimentally. Additionally, rapid advancement in proteomics have steered researchers to organize the study of protein structure, function, relationships, and dynamics in space and time. Thus the effective integration of all of these technological interventions will eventually lead to steering up of next-generation systems biology, which will provide valuable biological insights in the field of research, diagnostic, therapeutic and development of personalized medicine.

## Introduction

The emergence of “innovative biology” is accompanied by the birth/innovation of other sciences, such as computational biology and bioinformatics, which have a combined interface of molecular biology. Due to the large datasets generated, its management and storage become critically important. Therefore, different databases came into existence, which organise a large amount of biological information stored and processed to permit the scientific community access (Ritchie et al., 2015). The increasing amount of data has been abetted by an increase in the number of biological databases (Pevsner, 2015). Usually public databases accumulate big amounts of information, and they are categorised into primary and secondary databases. The primary databases are composed of the findings of experimental data that are reported without any critical analysis related to previous publications (Luscombe et al., 2001; Prosdocimi, 2010). However, in the secondary databases, there is a collection and explication of data, called process of content curation. Besides various functional databases such as the Kyoto Encyclopedia of Genes and Genomes (KEGG) and Reactome that allow analysis and explanation of metabolic maps. Various primary databases like DNA Database of Japan (DDBJ), GenBank at the National Center for Biotechnology Information (NCBI), and European Molecular Biology Laboratory (EMBL) remained as the main databases of nucleotide sequences and proteins. International Nucleotide Sequence Database Collaboration (INSDC) being the parent organisation of these databases and sharing among each other the deposited information daily (Prosdocimi et al., 2002; Amaral et al., 2007; Pevsner, 2015).

Last 2 decades have witnessed great advancements in molecular biology, data analysis procedures were established at a fast pace to enable the interpretation of the large amount of information produced mainly by DNA sequencing technologies that produced the exponential amelioration of genomics, transcriptomics and proteomics information. Biological data of genomics/proteomics although considered to be the recent domains, have emerged interdependently and created a historical impact on the available information coupled with innovations in computational resources, resulted in huge biological data and data analysis that can enhance and intensify the developments in medical science (Verli, 2014). In the current modern times ‘-omics’ suffix include the genomics, transcriptomics, proteomics, phylogenomics, metabolomics and metagenomics, associated with large-scale biological data and the allied bioinformatics analysis. The emergence of newest high-throughput sequencing innovations, starting with improvements in Sanger sequencing, innovations in NGS technologies and next-generation proteomics, resulted in emergence of novel findings in the clinical settings (Zhou et al., 2010).

## Genome-Wide Approach—From Genome to Proteome

DNA sequencing plays a crucial role in the progression of molecular biology, not only changing the genetic landscape of genome designs but also opening up new opportunities in therapeutic arena and personalised medicine

## Genomics

Generally, Genomics is the domain that aims to uncover and explore structure, function, and innovative realm of genomes applying bioinformatics tools to explore sequenced genomes. (Altmann et al., 2012).

**FIGURE 1:**
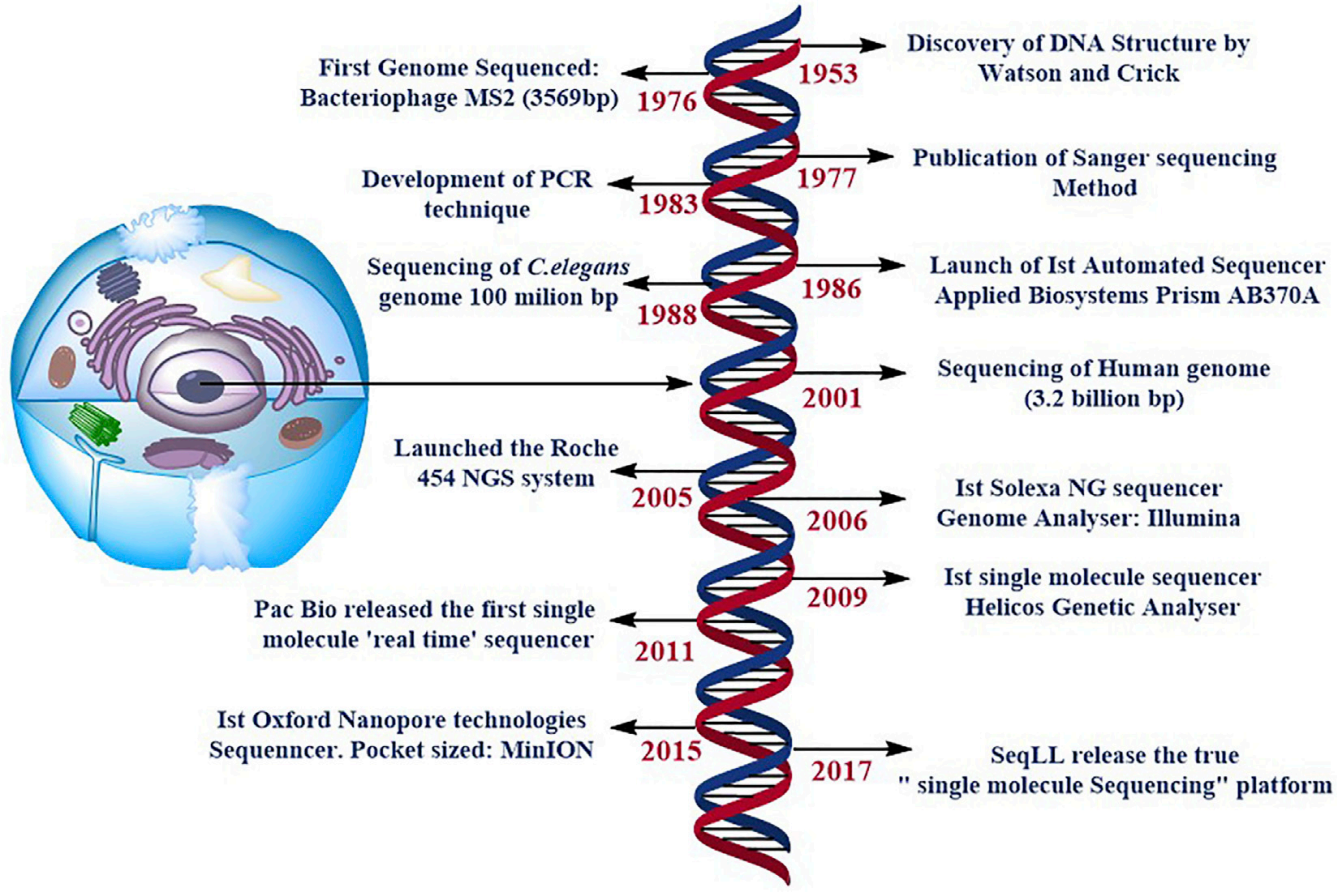
specifies the timeline of DNA sequencing. Some of the most significant and ground-breaking developments in DNA sequencing. NG stands for next generation, and PCR is for polymerase chain reaction. SMS stands for single molecule sequencing, and SeqLL stands for sequence the lower limit.

Paul Berg’s (Jackson et al., 1972), Frederick Sanger’s (Sanger and Coulson, 1975), and Walter Gilbert’s (Maxam and Gilbert, 1977) pioneering work on DNA sequencing enabled several developments, including the advances that opened up completely new potentials for DNA analysis, Sanger’s ‘chain-termination’ sequencing technology, more commonly known as Sanger sequencing (Sanger et al., 1977). Further technological advancements steered in the rise of DNA sequencing, led to the development of the first automated DNA sequencer (ABI PRISM AB370A) to be released in 1986, allowing drafting of the human genome to be completed during the next decade (Venter et al., 2001). These new methods are meant to supplement and eventually replace Sanger sequencing Figure 1. This technology is commonly known as next-generation sequencing (NGS) or massively parallel sequencing (MPS), which encompasses a wide range of methodologies. It is feasible to create huge amounts of data & each instrument runs in a faster and more cost-effective manner using this technology. The Next Generation Sequencing market is currently developing and expanding, with the world-wide market expected to reach 21.62 billion US dollars by 2025, up around 20% from 2017 (BCC Research, 2019). As a result, multiple brands are currently competing in this business, including BGI Genomics, Illumina, Ion Torrent (Thermo Fisher Scientific), PacBio and Oxford Nanopore Technologies etc. All of them provide distinct approaches to the same query: the generation of sequencing data. Second-generation sequencing relies on large parallel and clonal amplification of molecules (PCR, polymerase chain reaction) (Shendure and Ji, 2008), whereas third-generation sequencing depends on sequencing of single-molecules without a preceding clonal amplification (Schadt et al., 2010; van Dijk et al., 2018; Ameur et al., 2019). Although the process of NGS include various steps:

1) **NGS library Preparation:** A library comprises DNA/RNA fragments that denotes the full genome/transcriptome or a region of interest in next-generation sequencing. Each NGS platform has its own unique features, in general, the production of an NGS library begins with fragmentation of the DNA/RNA, followed by the connection of sequence adaptors to fragments to permit enrichment of those fragments. The sensitivity and specificity of a good library should be high. This implies that all relevant fragments should be properly represented in the library and that there should be no random errors (non-specific products). It is easier said than done, though, because genomic areas are not all equally susceptible to sequencing, making the creation of a sensitive and specialised library difficult and cumbersome (Aird et al., 2011).

2) **NGS Platforms**

Platforms for Second-Generation Sequencing

The category of cyclic-array sequencing technologies (Amaral et al., 2007) includes second-generation systems. The production and library amplification (made from RNA/DNA samples), clonal growth, sequencing, and investigation are all part of the core workflow for second-generation platforms. Ion Torrent and Illumina are the two most well-known sequencing firms for second-generation sequencing systems (Kircher et al., 2011; Quail et al., 2012).

3) **Platforms for Third-Generation Sequencing:**

The ability to avoid limitations of PCR-based methods, such as nucleotide misincorporation by a polymerase, formation of chimaera and drop-outs of alleles resulting in an false homozygosity call, was made possible by 3^rd^-generation NGS technology (Thompson and Steinmann, 2010). The Helicos Genetic Analysis System was the first commercial third-generation sequencer (Pushkarev et al., 2009). The Pacific Biosystems (PacBio RS II sequencer) established the notion of single-molecule real-time (SMRT) sequencing in 2011 (McCarthy, 2010). Furthermore, this method allows for the sequencing of lengthy reads (up to 30 kb on average). Individual DNA polymerases are coupled to zero-mode waveguide (ZMW) wells, which are nanoholes where a single DNA polymerase enzyme molecule can be put directly (McCarthy, 2010). PacBio has released the Sequel II System, which claims to cut project costs and timelines by up to 175 kb with highly accurate individual long reads (HiFi reads) compared to previous versions (Pereira et al., 2020).

Merker and co-workers demonstrated initially to use a PacBio System for sequencing of long-read genomes to find a pathogenic variant in Mendelian disease patients, indicating that this method has a lot of potential for identifying structural variation (Merker et al., 2018). The Chromium instrument, which uses gel beads in emulsion (GEMs) technology, was released by 10X Genomics in 2016 (Pereira et al., 2020). The benefit of GEMs technology is that it cuts down on time, beginning material, and prices (Zheng et al., 2016; Zheng et al., 2017; Pereira et al., 2020). With low false positives and high throughput, the chromium system can also perform single-cell genomic and transcriptional profiling, immunological profiling, and chromatin accessibility studies at single-cell resolution. As a result, intriguing new applications are emerging, particularly in the areas of epigenetics research, *de novo* genome assembly, and long sequencing reads (Delaneau et al., 2019; Laurentino et al., 2019; Wang et al., 2019).

**4) Innovative Bioinformatics approach:** Sequencing platforms are improving, and it is now possible to sequence the human genome in as little as a week or two. Thus, the huge data generated necessitates bioinformatics and computational expertise to organise, analyse, and infer NGS data. As a result, NGS bioinformatics is undergoing significant development, which can only be aided by improving computational capabilities (hardware) as well as algorithms and applications (software) to streamline all required steps: from processing of raw data to detailed data analysis and variant interpretation in a clinical setting.

**Analysis of the NGS data:** NGS bioinformatics is usually classified into three categories: primary, secondary, and tertiary analysis (Pereira et al., 2020).

The primary data analysis includes the identification and evaluation of raw data (signal analysis), the target of the generation of legible sequencing reads (base calling), and the estimation of base quality (Ledergerber and Dessimoz, 2011). This main analysis often produces a FASTQ file (Illumina) or an unmapped binary alignment map (uBAM) file (Ion Torrent).

Secondary analysis, which involves read alignment against the reference human genome (usually hg19 or hg38) and variant calling, is the next step in the NGS data analysis workflow.

Read alignment, which includes aligning sequenced fragments (processed data) against a reference genome, or *de-novo* assembly, which involves constructing a genome from basic without the use of external data, are two options for mapping sequencing reads. The availability or absence of a reference genome could be enough to decide between one technique and another. Nonetheless, reference sequence mapping is the preferred method for most NGS applications, particularly in clinical genetics (Flicek and Birney, 2009). However, *de-novo* assembly, on the other hand, is primarily limited to more focused tasks, such as correcting flaws in the reference genome and improving the detection of SV and other complicated rearrangements and newer findings (Ameur et al., 2018).

**FIGURE 2:**
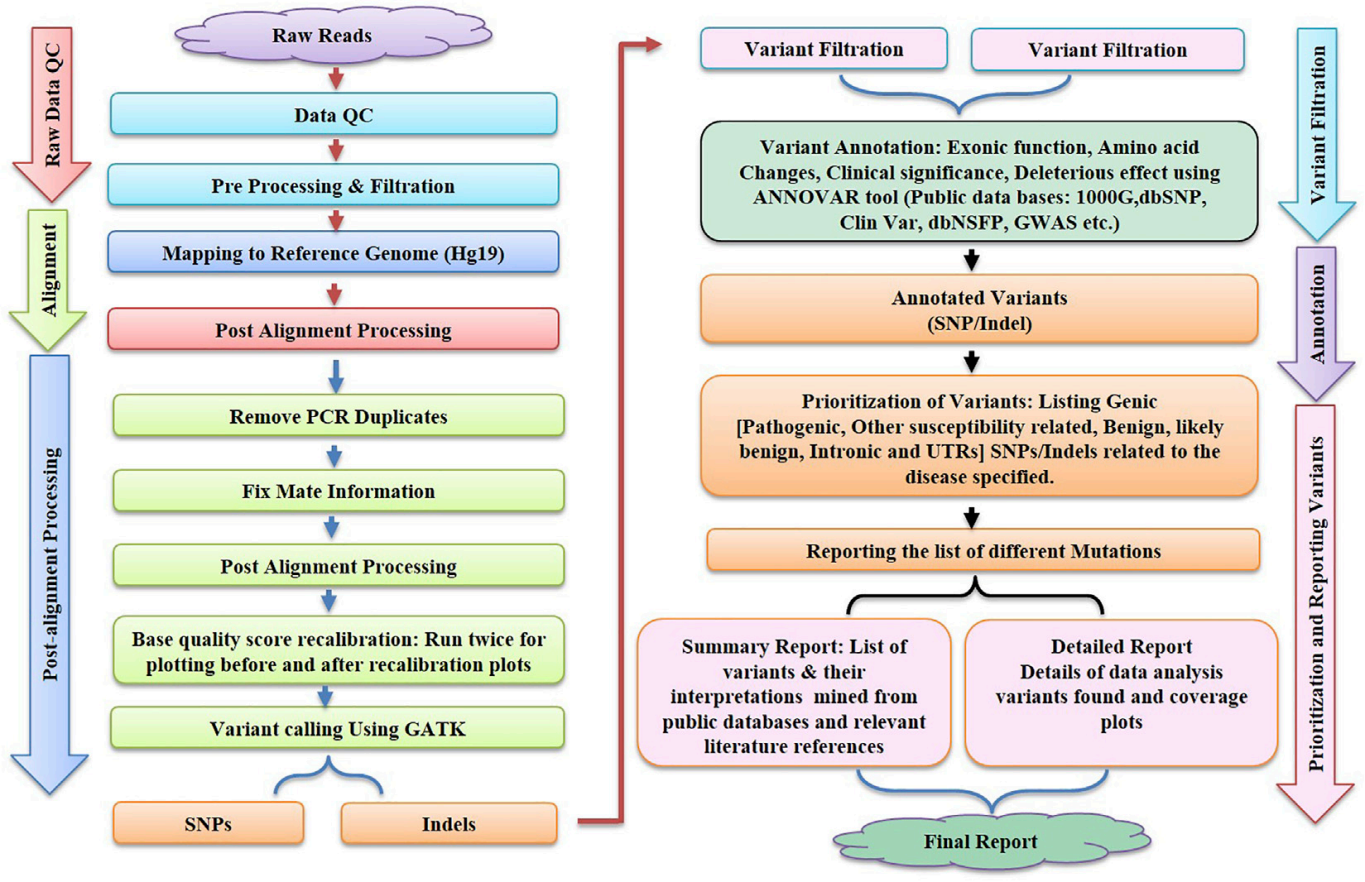
illustrates the various steps like Raw Data Quality Control, Alignment, Post Alignment Processing, Variant Filtration, Annotation and Reporting of variants involved in bioinformatics workflow for next-generation sequencing (NGS).

In the context of human clinical genetics, the third main phase of the NGS analysis pipeline addresses the essential issue of “making sense” or data interpretation, which requires finding the basic link between variant data and the observed phenotype in a patient. The tertiary analysis starts with variant annotation, which adds a fresh layer of data to predict the functional impact of all variants found during the variant calling procedure. Variant filtering, prioritisation, and data visualisation approaches are utilised after variant annotation. These procedures can be carried out utilising a number of software suites, which must be updated on a regular basis to reflect the most recent scientific findings, necessitating ongoing maintenance and development on the part of the developers. The generalised workflow of NGS is shown in Figure 2 **.**

Variant annotation is a crucial first step in the assessment of sequencing variants. As previously indicated (Scherer et al., 2007), variant calling generates a VCF file. Each line in such a file contains high-level information about a variant, such as genomic position, reference, and alternate bases, but no information biological implications. Variant annotation provides biological context for all discovered variants. Data annotation is performed automatically due to the large amount of NGS data. For variant annotation, several programmes are currently available, each of which uses distinct approaches and databases such as Sorting Intolerant from Tolerant (SIFT), (Ng and Henikoff, 2003), PolyPhen-2, (Adzhubei et al., 2010), Combined Annotation Dependent Depletion (CADD) (Kircher et al., 2014) and Condel (González-Pérez and López-Bigas, 2011), compute the impact scores for each variant based on various specifications, such as sequence homology, conservation of amino acid residues, evolutionary conservation, structure of protein, or statistical prediction based on known mutations, are integrated into such annotation tools. Furthermore, annotation can be used to search disease variant databases like ClinVar and HGMD for information on their clinical associations. Annotate Variation (ANNOVAR) (Yang and Wang, 2015) variant effect predictor (VEP) (McLaren et al., 2010), Single Nucleotide polymorphism effect (snpEff) (Cingolani et al., 2012), and SeattleSeq (Ng et al., 2009) are the most extensively used annotation tools among the many available. SNPs, INDELs, and Copy Number Variation (CNVs) can all be found using ANNOVAR, a command-line tool. It compares variants and explicates the functional consequence of variants on genes and other genomic components (Wang et al., 2010a). The overall number of variants obtained after analysis of a VCF file from WES may range between 30,000 and 50,000. Filtering algorithms are required to find the variant(s) responsible for a particular disorder. Some more examples in Table 1. As a result, it is strongly advised to eliminate false-positive calls and variant call errors when beginning the third level of NGS analysis, depending on quality parameters or prior knowledge of artefacts. The population frequency filter is one of the most widely used NGS filters. One of the filter based on allele frequency is minor allele frequency (MAF), which can sort variations into different categories: uncommon variants (MAF 0.5, usually picked for Mendelian illnesses), low frequency variants (minor allele frequency between 0.5 and 5%), and common variants (MAF >5%) (Consortium et al., 2010). It not only aids in better identifying disease alleles, but also in understanding population migrations, relationships, origins, admixtures, and population size changes, which may be useful in understanding various disease patterns (Stoneking and Krause, 2011). The most extensively utilised databases are the 1,000 genome project (Siva, 2008), Exome Aggregation Consortium (ExAC) (Lek et al., 2016), and the Genome Aggregation Database (gnomAD; http://gnomad.broadinstitute.org/). This filter, however, has limits and may result in incorrect exclusion.

**TABLE 1:** Demonstrates a list of commonly used tools for performing an NGS functional filter, along with examples.

S.No	Software	Description	Ref
1	**Phylo PPhylogenetic** *p* **-values**	The patterns of conservation (positive scores)/acceleration (negative scores) for various annotation classes and clades of interest are investigated using a neutral evolution model	Pollard et al. (2010)
2	**SIFT Sorting Intolerant from Tolerant**	Based on the sequence homology, Predicts whether an AA change would affect protein function and maybe alter the phenotype. A variation with a score of less than 0.05 is considered deleterious	Ng and Henikoff, (2003)
3	**PolyPhen-2 Polymorphism Phenotyping v2**	Using a naive Bayes classifier, predicts the functional impact of an AA substitution based on its individual properties Two tools are included. HumDiv (intended for use in complicated phenotypes) and HumVar (designed for Mendelian disease diagnosis). Higher scores (>0.85) predicts more confidently, damaging variants	Adzhubei et al. (2010)

4	**CADDCombined Annotation Dependent Depletion**	Scores all human SNV and Indel using a combination of genomic annotations. According to functional categories, effect sizes, and genetic architectures, it prioritizes functional, deleterious, and disease-causing variations. Pathogenic variants should be identified using a cut-off score of 10 or above	Kircher et al. (2014)
5	**MutationTaster**	Evaluates evolutionary conservation, splice-site alterations, protein loss, and changes that could affect mRNA levels. Polymorphisms and disease-causing variants are both classed as polymorphism	Schwarz et al. (2010)
6	**nsSNPAnalyzer**	Extracts structural and evolutionary information from a query nsSNP and predicts its phenotypic effect using a machine learning method (Random Forest). The variant is divided into two categories: neutral and disease	Bao et al. (2005)
7	**TopoSNP Topographic mapping of SNP**	SNPs are analysed based on their geometric position and conservation information, resulting in an interactive visualisation of disease and non-disease linked with each SNP.	Stitziel et al. (2004)
8	ANNOVAR * Annotate Variation	Annotates variants based on a variety of criteria, including whether SNPs or CNVs affect protein function (gene-based), locating variants in specified genomic regions outside of protein-coding regions (region-based), and locating known variants in public and licensed databases (filter-based)	Yang and Wang, (2015)
9	VEP *Variant Effect Predictor	Determines the impact of numerous variants (SNPs, insertions, deletions, CNVs, or structural variants) on genes, transcripts, and protein sequences, as well as regulatory domains, on genes, transcripts, and protein sequences	McLaren et al. (2010)
10	snpEff *	SNV are annotated and classified based on their effects on annotated genes, such as synonymous/nsSNP, start or stop codon gains or losses, genomic positions, and so on Considered a structurally based annotation tool	Cingolani et al. (2012)
11	**SeattleSeq**	Provides dbSNP rs IDs, gene names and accession numbers, variant functions, protein locations and AA changes, conservation scores, HapMap frequencies, PolyPhen predictions, and clinical association for SNVs and tiny indels	Ng et al. (2009)

The bold values are the names of software/tools.

Even though, functional annotation offers a significant information for filtering, the most critical question to answer, especially in the context of gene discovery, is whether a given variant or mutant gene the disease-causing gene? What is its frequency in different population sets studied globally? To solve this difficult issue, a new generation of tools is being created that, rather than just omitting information, rate variants and allow them to be prioritised. (MacArthur et al., 2012; Lelieveld et al., 2016; Harper, 2017). Various ways have been suggested e.g. PHIVE investigates the similarities between human illness phenotypes and those derived from animal model organism knockout experiments (Robinson et al., 2014). While other methods try to handle the problem in a novel way, by computing a lethal score (also known as burden score) for each gene using data from population variation databases (Eilbeck et al., 2017).

Phevor, which uses data from other relevant ontologies, such as gene ontology (GO), to advocate novel gene–disease connections, can also be employed for the identification of novel genes (Singleton et al., 2014). The fundamental purpose of these tools is to provide a small number of variants that can be validated using molecular techniques (Pereira et al., 2019a; Pereira et al., 2019b). VarSeq/VSClinical (Golden Helix), Ingenuity Variant Analysis (Qiagen), Alamut^®^ software (interactive biosoftware), and VarElect have all recently been developed commercial softwares for the elucidation and prioritisation of variants in a clinical context, to be used by clinicians, geneticists, and researchers (Stelzer et al., 2016). Apart from the tools that aid in variant analysis and elucidation, clinicians now have access to medical genetics firms like Invitae (https://www.invitae.com/en/) and CENTOGENE (https://www.centogene.com/) that provide a precise medical diagnosis.

**5) Third generation sequencing technologies** has the capability of sequencing single molecules with average read lengths of >10,000bp -100,000bp or even more. The advent of this technology has eliminated the requirement of amplification of DNA (PCR) and it provides real time results (Pereira et al., 2020). The third-generation sequencing services are provided by Pacific Biosciences (PacBio) that utilizes the single molecule real time (SMRT) platform and fluorescent nucleotide detection methodology. Oxford Nanopore Technologies (Minion) which utilizes the nanopore methodology where an ionic current passes through the flow cell and nucleotides bases are determined by the changes they produce in the current respectively when pass through the nanopores. (Xiao and Zhou, 2020).

The bioinformatic tools required to analyze the data obtained from the third-generation sequencing technologies needs to be more specific and error prone. Some tools are depicted in Table 2.

**TABLE 2:** Demonstrates various software used in third generation sequencing.

S.No	Software	Description	Ref
1	MinHash Alignment Process (MHAP)	Detects long read overlaps	Berlin et al. (2015)
2	Minimap/miniasm	*De novo* assembler for long reads	Li, (2016)
3	DALIGN	finds overlaps and local alignments in very noisy long read DNA sequencing data sets	Li, (2016)
4	Graphmap	detects single-nucleotide variant calling on the human genome; have increased sensitivity of 15%; provides precise detection of structural variants from length 100 bp - 4 kbp	Sović. (2016)
5	BLASR	Maps long reads influenced by insertion and deletion errors	Chaisson and Tesler, (2012)
6	Nanocorrect	Error correction in long reads	Loman et al. (2015)
7	PBJelly	For gap closing in genome assembly	English et al. (2012)
8	HGAP	De novo assembly	Chin et al. (2013)
9	PoreSeq	Variant calling	Szalay and Golovchenko, (2015)
10	Nanocorr	Error correction/*de novo* assembly/*de novo* mutation or SNPs detection	Goodwin et al. (2015)
11	Nanocall	Variant calling	David et al. (2017)
12	DeepNano	Base caller	Boža et al. (2017)
13	Nanopolish	Enhances the base quality	Loman et al. (2015)

**Limitations:** Although Third generation sequencing technology is fast and provide real time result however still NGS are preferred as the error rate is less in NGS as compared to third generation sequencing which is ∼15%. Due to this high error rate, the technology can miss the detection of SNPs/point mutations and not best suited for mutational analyses. The methodology requires improvement. Moreover, there is need to develop more bioinformatic tools and algorithms for the downstream data analyses that is again a challenge for researchers for the time being (Ozsolak, 2012).

## Transcriptomics

cDNA sequencing or RNA-seq when compared to other methods allows for more accurate mapping of reads and quantification at the transcript level. Differential expression analysis and identification of isoforms due to mRNA splicing, NGS of **Small non-coding RNA** as well as the discovery and characterisation of novel transcripts, are examples of high throughput applications (Marioni et al., 2008; Wang, 2009; Montgomery et al., 2010).

**Small non-coding RNA NGS:** A significant increase has been seen in the research community related to biomarkers which aids in the prediction, early detection and prevention of the disease. The biomarkers research helps the scientific and clinical community significantly in improving the clinical outcomes (Lopez et al., 2015). Non-coding RNAs (ncRNAs) have become the biomarker hotspot of the research interest in the field of disease identification and treatment. MicroRNAs (miRNAs) are the type of ncRNAs which are mostly explored for their potential biomarker role (Lopez et al., 2015). Till date ncRNA studies have been performed mainly by qRT-PCR, *in situ* hybridization, or microarray techniques. NGS has opened a new way to analyze/detect the RNA molecules present in the biological samples. NGS tenders several methodological advantages over other technologies like increased throughput, decreased RNA input, good consistency and quality of data, higher detection depth, analysis of all RNA populations, and discovery of novel molecules (Liu et al., 2021). A typical RNA-sequencing experiment consists of the following steps:

Thus all the above possibilities have allowed us to learn more about the genome’s organisation, the molecular constituents of cells and tissues, and the complexities of regulatory systems (Zhou et al., 2010; Sims et al., 2014). Many investigations, both fundamental and applied, have focused on mRNA splicing. Between the transcriptional and translational level, splicing occurs in every eukaryotic cell. Pre-mRNA transcripts may be variably spliced depending on location of tissue and/or stage of development, allowing multiple transcripts to be generated and hence distinct proteins to be made from the same gene (Burge et al., 1999; Nilsen, 2003). The divergence of splice site sequences from the prototypes has been linked to the generation of alternative transcripts. Furthermore, in most introns of higher eukaryotes, these extremely degraded motifs may be observed. Pseudo-exons are intronic sequences of standard exon size that outnumber real exons and are flanked by sequences that fit the exon’s 5′ and 3′ splicing signal requirements, but are never recognized as proper exons by the spliceosome. To distinguish true exons and splice sites from pseudo exons, splicing machinery must rely on auxiliary sequence features such as intronic and exonic cis-elements (Jacob and Gallinaro, 1989).

Exonic Splicing Enhancers (ESEs) are the most researched and well explored among them. They’re nucleotide sequences of short length that are primarily targeted by Serine/Argine-rich (SR) proteins, which then help to define exons (Blencowe, 2000). Exonic Splicing Silencers (ESSs), on the other hand, assist the spliceosome in neglecting pseudo exons and decoy splice sites. They serve as binding sites for exon exclusion-promoting proteins (mostly hnRNP proteins) (Zhu et al., 2001). Several bioinformatics approaches have been created and are now accessible to examine or predict splice signals (Zhang et al., 2005). One of the most essential bioinformatics tools is HSF (Human Splice Finder). For administration of data, designing of algorithm and online interface, HSF was built with the 4D package (4D S.A.). The HSF database was created with all human genes containing introns and exons. It was created using an Ensembl dataset that included about 22 000 genes and 46 000 transcripts from *Homo sapiens.* Because matrices and methods were specifically built for the human genome, the HSF database exclusively contains human genes (Flicek et al., 2008). HSF also has data taken from the Ensembl Variation Database (EVD), which can be used to investigate the impact of SNPs on splicing. A Perl script was written utilizing the Ensembl Perl API to allow HSF to access the EVD directly and get SNPs in human genes. Because matrices and methods were specifically built for the human genome, the HSF database exclusively contains human genes (Flicek et al., 2008).

**FIGURE 3:**
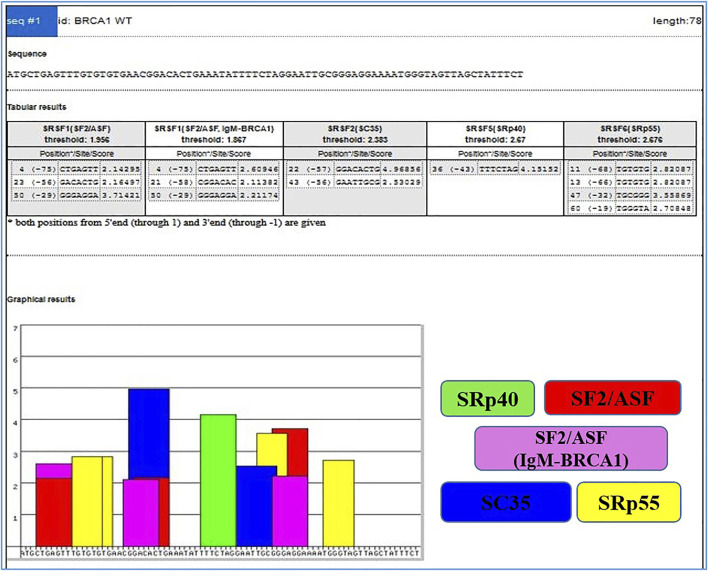
Output window with complete list of scores. High scores are represented as color-coded bars. The height of each bar indicates the score value (motif score), and its width and placement on the *x*-axis represent the length of the motif (6–8 nt) and its position along the sequence.

On the other hand, Exonic splicing enhancers (ESEs) can be disrupted by nonsense, missense, and even translationally silent mutations, causing the splicing machinery to skip the mutant exon with significant consequences on gene structure. The frequency of mutations, whose major consequence is unusual splicing has been significantly underestimated because the effects of mutations are most often predicted purely based on information of genomic sequence (Cartegni et al., 2002). ESEs are found in both alternative and constitutive exons, where they serve as binding sites for Ser/Arg-rich proteins (SR proteins), a family of conserved splicing factors involved in a variety of splicing stages (Graveley, 2000). Through their RNA-binding domain, SR proteins promote exon definition by attracting spliceosomal components via protein–protein interactions facilitated by their RS domain and/or antagonizing the function of surrounding splicing silencers. Multiple categories of ESE consensus motifs have been described, and different SR proteins have varying substrate specificities (Graveley, 2000; Cartegni et al., 2002; Fairbrother et al., 2002). Using weight matrices for four different human SR proteins, ESE finder searches query sequences for potential ESEs. The matrices are based on frequency values produced from the alignment of winning sequences obtained through functional SELEX studies, corrected for the background nucleotide frequency of the initial SELEX library, which was created using chemical synthesis (Liu et al., 1998; Liu et al., 2000). The query sequences can be entered directly into the input box or submitted as a text file. Multiple sequences can be processed at the same time if they are preceded by a FASTA-format description line (starting with ‘>’). Despite the fact that ESEfinder is a tool for RNA analysis, it only accepts normal DNA nomenclature (A, C, G, and T, not U). Any character other than the letters A, C, G, and T, as well as spaces and paragraph breaks, will be ignored by the programme. Although both upper and lower case are acceptable, the output lines will be written in upper case. The user can choose from one to four matrices to be used at the same time. The result for each matrix is a series of 1 ntd incremented scores. Only the ‘hits’ or ‘high score motifs’ are displayed in the initial output window, Figure 3 which include the position of the first nucleotide, the motif match sequence, and the calculated score. When a score exceeds the threshold value set in the input page, it is deemed a high score.

By choosing the ‘custom’ button and entering the required value into the box, any score can be used as the cutoff threshold. As a result, ESEfinder may be used to identify potential ESEs, and the prime application is the accurate interpretation of the impact of disease-associated variants. It has been previously demonstrated that ESEs predicted by this matrix-based method cluster in places where natural enhancers have been empirically localized and are more common in exons than in introns (Cartegni et al., 2003).

**CircRNAs:** In contrast to messenger RNAs, circular RNAs (circRNAs) are physiologically active nucleic acid molecules that occur in closed loop RNA forms and do not have polyadenylated tails. CircRNAs are classified as non-coding RNA (ncRNA), yet some circRNAs have the ability to code for proteins. CircRNAs were originally discovered and identified in plant viroids in the 1970s, and then in the cytoplasm of eukaryotic cells in the 1980s. Due to the prevalence of linear RNAs, early development in this field was likely modest, and circRNAs were thought to be a consequence of RNA splicing. Recent advancements in next-generation sequencing and related bioinformatics technologies, on the other hand, have speed up research in humans, mice, nematodes, plants, and archaea have all been found to have these compounds (Chen et al., 2021). Various tools employed for the analysis of circRNAs are summarized in Table 3, below.

**TABLE 3:** Showing the various bioinformatic software tools used in circRNAs analysis.

Tool name	TT	Installation Type	ATMR	PL	CV	Platform	Ref
CIRCexplorer	De novo; annotation	pip, Conda, Docker	STAR, BWA	*Python *	v2.3.8	Unix/Linux	(Zhang et al., 2014a)
CircPro	De novo; annotation	MID	BWA (CIRI2)	Perl	—	Unix/Linux	Meng et al. (2017)
MapSplice	De novo; annotation	Conda	Bowtie	*Python *	v2.2.1	Unix/Linux	Wang et al. (2010b)
circRNA_finder	De novo	MID	STAR	Perl, AWK	v1.2	Unix/Linux	(Westholm et al., 2014; Jia et al., 2019)
CircRNAFisher	De novo	MID	Bowtie2	Perl	v0.1	Unix/Linux	Westholm et al. (2014)
miARma	De novo	Docker, Virtual box image	BWA (CIRI)	Perl, *Python*, R	v1.7.5	Unix/Linux, Windows	Andrés-León et al. (2016)
CIRI	De novo	MID	BWA	Perl	v2.0.6	Unix/Linux	(Gao et al., 2015; Gao et al., 2018; Zheng et al., 2019)
ACFS	*De novo*	MID	BWA BLAT	Perl	v2.0	Unix/Linux	You and Conrad, (2016)
CircDBG	Annotation	CR	k-mer (no need aligner)	C++	-	Unix/Linux	Li and Wu, (2020)

Header Abbreviations: TT, tools type; IT**,** installation type; CV, current version; Ref, reference; ATMR, aligner or tools or method required; PL, programming language.

## Proteomics

**FIGURE 4:**
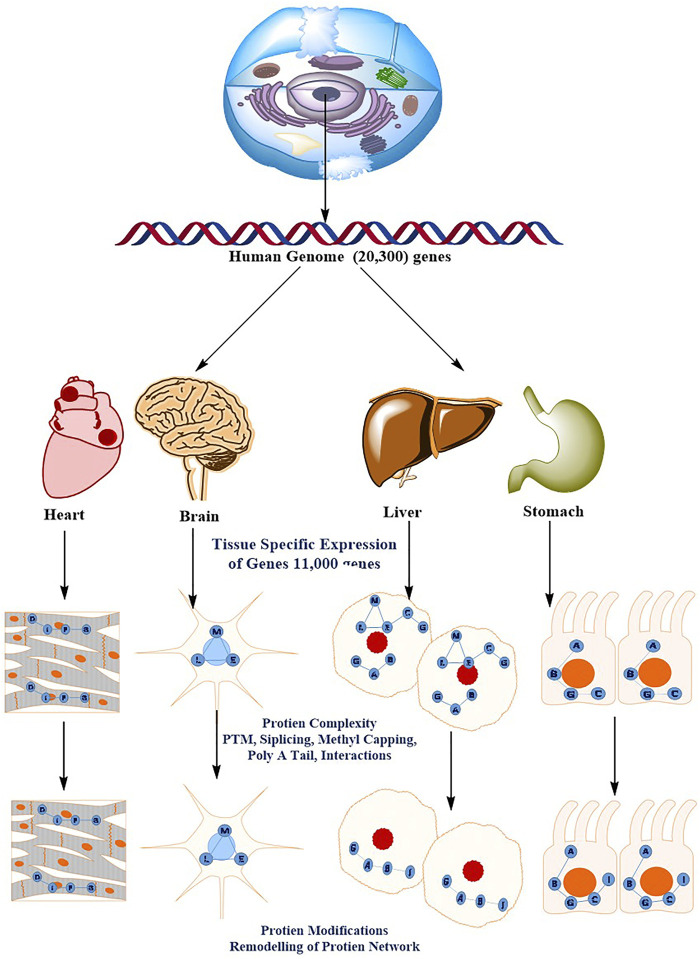
The diverse and dynamic methods of proteome regulation give the human genome a higher level of complexity. There are roughly 20,300 genes in the human genome. The molecular basis of the cellular phenotype (that is, the tissue cell types) is determined by the specific expression of a subset of the genome (11,000 genes). The sophisticated methods of protein regulation, such as splicing variations PTMs, post-translational modifications; PPIs, protein–protein interactions, and subcellular localization, acquire a considerably higher order of complexity. This results in tissue- and organelle-specific protein networks that respond to perturbations differently throughout time (for example, ageing or drug treatment).

Understanding the molecular processes that mediate cellular physiology requires the identification, quantification, and characterization of a cell’s whole protein content (Schmidt et al., 2014; Jensen et al., 2006). A rapid advancement in proteomics has steered the researchers to organize the study of protein structure, function, relationships, and dynamics in space and time. The groundbreaking revelation that DNA contains all of the genetic instruction required to build an organism gave rise to molecular biology’s central dogma, which characterized a one-way flow of information from DNA to RNA to Proteins. This belief has been debunked by recent discoveries. Epigenetic markings, alternative splicing, non-coding RNAs (including microRNAs), protein–protein interaction (PPI) networks, and post-translational modifications (PTMs) are only a few examples of how genotype and phenotype are not solely determined by information on the genome (Nagaraj et al., 2011; Beck et al., 2011; Baker, 2012). Proteomics is the global study of proteins, which are the key functional entities in the cell. This analysis is arguably the most important level of information required to understand how cells work. When compared to data collection at the genomic and transcriptomic levels, the proteomic data acquisition has proven difficult. Global protein analysis is a difficult analytical task, in part because amino acids, the building blocks of proteins, have such a wide range of physicochemical properties. Furthermore, in comparison to the genome, the proteome is enriched by alternative splicing and a wide range of protein modifications and degradation, and the complexity is heightened by the interconnectivity of proteins into complexes and signaling networks that are highly divergent in time and space Figure 4 (Cox and Mann, 2011). A decade ago, sequencing and identifying a single protein was a big problem; however, today’s high-throughput technology allows for the identification and quantification of essentially all expressed proteins in a single experiment. Similarly, 10 years ago, MS-based phosphoproteomics could only identify a few hundred phosphosites, whereas currently more than 30,000 phosphosites can be quantitatively monitored. This current method is referred to as “next-generation proteomics” to reflect its ability to characterize practically the whole proteome as a result of advancement in technology. Proteomics technologies, particularly MS-based Protein identification has advanced tremendously in recent years as a result of cumulative technological breakthroughs in instrumentation, sample preparation and computational analysis (Ficarro et al., 2002; Lemeer and Heck, 2009; Lundby et al., 2012).

Proteomics using mass spectrometry (MS) generates a large quantity of information about the expression, post-translational modifications (PTMs), and interactions among thousands of proteins. The obtained data must be supplied to the scientific community in a format that is both suitable and curated, as well as retrievable and interpretable. Proteomics data will be made freely available to the public, ensuring that quality standards are maintained in the area. The long-term storage of unprocessed raw data is a first level of distribution for proteomics data. Understanding the proteome’s complex and dynamic interactions necessitates the creation of physical interaction charts.

Proteins frequently interact with one another in stable or transient multi-protein complexes of varying composition, with the human interactome containing an estimated 130,000 binary interactions, the majority of which have yet to be mapped. Proteins can also interact with other molecules like RNA, DNA and metabolites. These complexes play crucial roles in regulatory processes, signalling cascades, cellular functions, and their inability to interact can result in their function being lost (Altelaar et al., 2012; Ma and Johnson, 2012). Tranche is one of the few public repositories that can manage this type of data at the moment, and it is based on an encrypted peer-to-peer system that stores data in numerous servers across the world. Raw data, on the other hand, is in a closed format, which makes it difficult to share. As a result, attempts are being undertaken to standardise formats that preserve all necessary information (Smith et al., 2011). The European Bioinformatics Institute’s PRIDE database exhibits this determination, as it enables the for the storage of both conventional MS data formats (XML) and associated peptide and protein identifications. Furthermore, including additional data (such as species, fragmentation procedures, and proteases) allows for a global meta-analysis of proteomic data sets (Perez-Riverol et al., 2019).

Moreover, Protein sequence alignment compares two or more than two sequences and aids in the identification of homologous regions, visualizing the relationship among sequences with respect to evolution and structure. It plays a crucial role in bioinformatics and helps in the query and construction of databases, prediction of protein’s primary, secondary and tertiary structure and biological function and many more. Many platforms are developed to analyse the sequence alignment. Some of them are PROSITE, Pfam, BLAST, FASTA, Clustal omega, T-Coffee, MUSCA, ALIGN, DIALIGN, ProbCons, HMMER3 phmmer and many more (Pruess and Apweiler, 2003; Sievers et al., 2011; Singh et al., 2016a).

Protein structure prediction can be done using the ProtParam tool from ExPasy (Expert Protein analysis system) (Gasteiger et al., 2005). It helps in the primary structure prediction of protein and aids in the computation of physicochemical properties of a given protein. The parameters that can be computed include molecular weight, amino acid and atomic composition, isoelectric point, estimated half-life, grand average of hydropathicity (GRAVY) and more. To predict the secondary structure, many tools have been developed till now including Chow-Fasman algorithim—a statistical approach which is based on calculation of statistical propensities of each residuum to form an α-helix or β-strand, GOR, Jpred, etc. Similarly, for tertiary protein structure prediction, PHYRE2 (Protein Homology/analogY Recognition Engine) (Kelley et al., 2015) and I-TASSER are available (Yang et al., 2015).

Apart from above mentioned software suits, there are other tools which are helpful in addressing protein analysis. Some of them are mentioned in Table 4.

**TABLE 4:** Demonstrates the Protein sequence analysis tool.

S.No	Software	Description	Ref
1	Expasy	A molecular server dedicated to protein and nucleic acid sequence analysis	Gasteiger et al. (2003)
2	Frame plot	Protein coding region prediction in Bacterial DNA	Ishikawa and Hotta, (1999)
3	MPEx	Membrane Protein Explorer (MPEx) is a tool that uses hydropathy plots based on thermodynamic principles to explore the topology and other properties of membrane proteins	Snider et al. (2009)
4	Predict Protein	Predict Protein is an online service that analyses protein sequences and predicts their structure and function. Predict Protein offers numerous sequence alignments, PROSITE sequence motifs, low-complexity regions (SEG), nuclear localization signals, regions lacking regular structure (NORS), and secondary structure predictions after users submit protein sequences or alignments	Bernhofer et al. (2021)
5	ProDom	Pro Dom is a database of protein domain families built by grouping homologous regions. The recursive PSI-BLAST searches [ALTS2] are used in the ProDom construction technique MKDOM2. Non-fragmentary protein sequences from the SWISS-PROT and TrEMBL databases were used as the starting point	Bru et al. (2005)
6	Prot Scale	Prot Scale lets you compute and visualise the profile generated by any amino acid scale on a given protein. Each type of amino acid is assigned a number value on an amino acid scale	Gasteiger et al. (2005)
7	Sequence Manipulation Suite (SMS)	The Sequence Manipulation Suite is a set of JavaScript tools for generating, formatting, and analysing short DNA and protein sequences in BioSyn’s Gizmo Tools	Stothard, (2000)
8	Worldwide Protein Data Bank (wwPDB)	The wwPDB hosts a single Protein Data Bank Archive of macromolecular structural data that is freely and openly accessible to the entire world	Berman et al. (2007)

To study the post-translational modifications, tools like GlycoMod (Cooper et al., 2001), NetPhos (Trost and Kusalik, 2011), NetPicoRNA (Smits et al., 2013), FindMod (Gasteiger et al., 2003), ScanProsite (De Castro et al., 2006) and others are available online. For protein interaction analyses STRING can be used (Szklarczyk et al., 2021). To visualize the 3-D structure of proteins, tools like Pymol and Jmol can be used. Pymol is also used to visualize the protein-ligand docking, binding site prediction, protein interactions and others (DeLano, 2002; Herráez, 2006).

**FIGURE 5:**
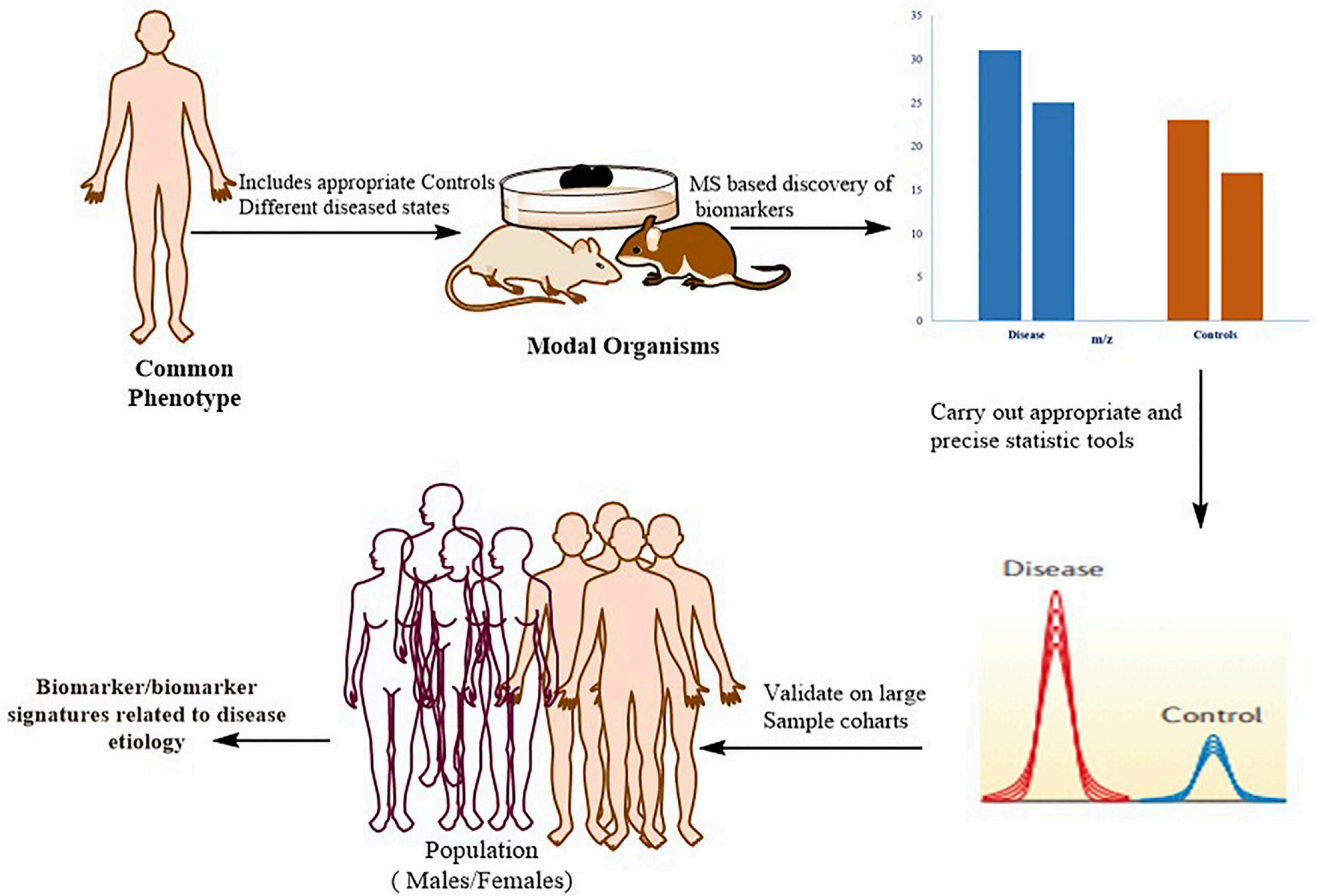
Typical workflow for identifying, validating, and stratifying protein-based biomarker signatures. Proteomics based on mass spectrometry (MS) is utilized for in-depth quantitative characterization of a disease model’s proteome and its appropriate control mechanisms. Following the application of strict statistics, a list of candidate proteins that can be used as a phenotypic signature is defined. These markers are verified in large patient cohorts using more specific methodologies, such as MS-based (for example, selective reaction monitoring (SRM)) or antibody-based approaches. To confirm that the biomarker has a direct mechanistic involvement in the disease, the biological connections between the signature proteins and the disease phenotype should be biochemically confirmed.

The identification of protein biomarkers with prognostic or diagnostic significance is one of the most difficult applications of proteomics right now Figure 5 **.**

As previously mentioned, recent technical advancements have resulted in the development of comprehensive pipelines that incorporate the discovery and validation phases, allowing plasma biomarkers to be identified for many diseases (Addona et al., 2011; Whiteaker et al., 2011). Despite the introduction of some successful biomarkers for clinical application, many (if not most) claimed biomarkers have weak reliability or lack rigorous confirmation, leading to scepticism among clinicians. The lack of proper controls in the discovery phase, the use of appropriate statistical tools for biomarker definition, and the need for independent validation steps in large patient cohorts to certify the legitimacy of the biomarker unambiguously are the primary flaws in many biomarker studies; such flaws lead to claimed biomarkers that are rarely directly related to disease biology (Poste, 2011).

### Metabolomics: Beacon for the 21st Century

After genomics, transcriptomics and proteomics, metabolomics is the innovative & newest of the “omics” sciences, combining high-throughput analytical techniques with bioinformatics. It is concerned with the quantitative and qualitative evaluation of metabolites, which are key metabolic intermediates and end products (Zhang et al., 2014b). The purpose of this scientific method is not only to figure out what pathological processes or disturbances are at the root of a specific disease entity, but also to anticipate how those conditions will respond to treatment interventions. Metabolomic analysis help discriminate between normal and abnormal pathways, which aids in disease diagnosis and prognosis prediction (Zhang et al., 2015). The potential of the metabolome to reflect environmental effects and to provide a snapshot of the individual’s pathophysiological status at a certain point in time is a noteworthy benefit of the metabolome over the genome (Shah et al., 2015; Zhang et al., 2015). The prime concern of the researchers/clinicians is the better understanding of the disrupted biochemical and pathological processes, as well as to inform the creation of more effective therapeutic medicines for the treatment of those illness states in humans. Metabolomic tools have the benefits of being quick, inexpensive, and sensitive. Metabolomics can be studied using a variety of techniques, including mass spectrometry (MS), nuclear magnetic resonance (NMR) spectroscopy, and Fourier-transform infrared (FTIR) spectroscopy. Metabolomic fingerprinting, metabolic profiling, metabolic footprinting, target analysis, and flux analysis are examples of such methods that all play important roles in understanding toxicological mechanisms and disease processes in live organisms (Tripathi et al., 2013; Zhang et al., 2013; Zhang et al., 2014b). Metabolomics is also critical in discovering new drugs, biomarkers for early disease diagnosis, such as rheumatoid or osteoarthritis (Carlson et al., 2018; Takahashi et al., 2019; Dudka et al., 2021), osteoporosis, cardiovascular disease, and Alzheimer’s disease (AD), cancer prognosis, diagnosis, and treatment (Pushkarev et al., 2009; McCarthy, 2010; Thompson and Steinmann, 2010; Kircher et al., 2011; Quail et al., 2012; Zheng et al., 2016; Zheng et al., 2017; Merker et al., 2018; Pereira et al., 2020), inborn errors of metabolism (IEM) and a variety of other applications (Carlson et al., 2018).

### Pharmacogenomics/Pharmacogenetics: *in-Silico* Approach

Pharmacogenomics is described as the study of genes and how medications alter an individual’s reaction. Pharmacogenomics is an emerging new discipline of science that combines pharmacology (the branch of science that studies drugs) with genomics (the branch of science that studies genes) to generate effective doses and safe pharmaceuticals tailored to an individual patient’s genetic makeup. One of the most important programs in which researchers are building and learning about genetic relationships and their impact on the body’s reaction to drugs is the Human Genome Project. Differences in genetic makeup influence pharmaceutical effectiveness, making it possible to anticipate medication effectiveness for an individual and investigate the presence of adverse drug reactions in the future (Caldwell et al., 2007).

Because of the wide range of individual responses to drug therapy, predicting the degree of effectiveness of a medication for a certain patient is difficult. Along with these clinical aspects, pharmacological factors such as variations in metabolism, drug distribution, and drug directed proteins play a significant role (Wattanachai et al., 2017). Table 5 describes various softwares employed in addressing Pharmacogenomics.

**TABLE 5:** Demonstrates various *in silico* approaches used in Pharmacogenomics.

S.No	Software name	Software Description	Ref
1	**Pharmacogenomics Knowledge (PharmGKB)**	It’s a comprehensive resource that compiles information on the impact of genetic variation on drug response, such as dosing guidelines, drug labels, gene-drug connections, and the genotype-phenotype link	Thorn et al. (2013)
2	**The Drug Gene Interaction Database**	DGIdb is a database and web interface for identifying drug-gene interactions, both known and unknown	Freshour et al. (2020)
3	**Side Effect Resource (SIDER 2)**	It covers data on marketed drugs and any adverse medication reactions that have been reported. Public documents and package inserts were used to gather the data. Side effect frequency, drug and side effect categories, and connections to additional information, such as drug–target relationships, are all included in the available data	Kuhn et al. (2016)
4	**Drug Bank**	Drug Bank Online is a comprehensive, free-to-use online database of drug and drug target information	Wishart et al. (2018)
5	**Search Tool for Interaction of Chemicals (STITCH)**	It uses data from the scientific literature and new research findings to describe chemical interactions with genes and proteins, as well as diseases and chemicals, and diseases and genes/proteins on humans	Kuhn et al. (2008)
6	**Genomics of Drug Sensitivity in Cancer**	The database contains data on the link between tumour cell genomes and anti-cancer drug sensitivity The sensitivity patterns of human cancer cell lines to a wide range of anti-cancer treatments were compared to genomic and expression data in order to find genetic factors that are predictive of sensitivity	Yang et al. (2013)

The bold values are the names of software/tools.

### Epigenomics—complex diseases: An enigma

Understanding the causes and mechanisms of complex non-Mendelian diseases remains a major issue and point of concern, despite substantial effort. Despite the fact that various molecular genetic linkage and association studies have been carried out in order to explain the heritable tendency to complicated disorders, the results are sometimes inconclusive and even contentious. Similarly, determining the environmental factors that cause a disorder is difficult (Singh Nanda et al., 2016). The emphasis is switched to epigenetic misregulation as a primary etiopathogenic element, which presents a novel interpretation of the paradigm of “genes plus environment”.

Various non-Mendelian irregularities of complex diseases, such as the presence of clinically indistinguishable sporadic and familial cases, sexual dimorphism, relatively late age of onset and peaks of susceptibility to some diseases, discordance of monozygotic twins, and major fluctuations on the course of disease severity, are consistent with epigenetic mechanisms. It is also been claimed that stochastic epigenetic processes in the cell may account for a significant percentage of phenotypic diversity formerly attributed to environmental factors. It is proposed that using epigenetic strategies in conjunction with traditional genetic strategies can greatly speed up the finding of etiopathogenic processes in complicated disorders (Lacal and Ventura, 2018). Epigenetic microarray technologies and *in silico* approaches will considerably enhance epigenetic investigations in complicated disorders as shown in Table 6.

**TABLE 6:** Showing various *in silico* approaches in Epigenomics.

S.No	Software name	Software Description	Ref
1	**DMRichR**	R package and executable for analysing and visualizing differentially methylated regions (DMRs) using CpG count matrices statistically (Bismarck genome-wide cytosine reports) It primarily employs the dmrseq and bsseq algorithms for upstream pre-processing, downstream analysis, and data display	Laufer et al. (2020)
2	**CpG_Me**	A whole genome bisulfite sequencing (WGBS) process for DNA methylation alignment and quality control that starts with raw reads (FastQ) and ends with a CpG count matrix (Bismark genome-wide cytosine reports)	Laufer et al. (2022)
3	**Rn Beads**	A Bioconductor (R) package for comprehensive analysis of DNA methylation data from Illumina Infinium arrays (450 K and EPIC) and BS-seq. MeDIP-seq and MBD-seq are also supported after some external processing	Müller et al., (2019)
4	**MEDIPS**	A Bioconductor (R) package for MeDIP (methylated DNA immunoprecipitation) and sequencing research (MeDIP-seq)	Lienhard et al. (2014)
5	**Minifi**	A Bioconductor (R) package for your Illumina Infinium arrays (450 K and EPIC) that enables complete analysis and takes cellular heterogeneity into account	Aryee et al. (2014)
6	**DMRcate**	A Bioconductor (R) package for the identification of DMR from the human genome using WGBS and Illumina Infinium array (450 K and EPIC) data	Peters et al. (2015)
7	**FEM**	Integrative analysis of DNA methylation and gene expression data	Gentleman et al. (2004)
8	**coMET**	Visualization of Epigenome-Wide Association Study (EWAS) from a genomic region	Martin, (2014)

The bold values are the names of software/tools.

### Pathway/Enrichment Analysis framework: omics Data

Comprehensive DNA, RNA, and protein quantification in biological materials is prevalent. The generated data is rapidly accumulating, and its analysis aids researchers in discovering new biological functions, genotype–phenotype correlations, and disease causes (Lander, 2011; Stephens et al., 2015). Many researchers, however, find that analysing and interpreting these data is a huge issue. Long lists of genes often emerge from analyses, requiring an impractically enormous amount of manual literature research to analyze.

Scientists can use pathway enrichment analysis to acquire mechanistic insight into gene lists generated by genome-scale (omics) investigations. This approach finds biological pathways that are more enriched in a gene list than is expected by chance (Nguyen et al., 2019). Innovative pathway enrichment analysis methodologies and provide a step-by-step guidance for interpreting gene lists generated by RNA-seq and genome-sequencing research. The approaches can be employed in various sets: defining a gene list using omics data, determining statistically enriched pathways, and visualizing and interpreting the results. This technique can be used in expressed genes and cancer genes that have been altered; however, the idea can be extended to a wide range of omics data (Paczkowska et al., 2020). Although there are various enrichment tools. Few of them are summarized in Table 7 **.**

**TABLE 7:** Showing various enrichment tools.

S.No	Software name	Software Description	Ref
1	**singular enrichment analysis (SEA)**	The enrichment P-value for each term from the pre-selected interesting gene list is calculated	Huang et al. (2009)
		Then, in a basic linear text style, the enriched terms are listed. The most traditional algorithm is this one The majority of enrichment analysis tools still rely on it	
2	**Gene set enrichment analysis (GSEA)**	The enrichment analysis takes into account all genes (without pre-selection) and their related experimental values. The following are the distinguishing characteristics of this strategy: I Unlike Classes I and II, there is no requirement to pre-select interesting genes; (ii) Experimental values are integrated into P-value computation	Subramanian et al. (2005)
3	**Modular enrichment analysis (MEA)**	This approach carries on the spirit of the SEA. The term–term/gene–gene associations, on the other hand, are taken into account when calculating the enrichment P-value The benefit of this technique is that the term–term/gene–gene interaction may contain biological meaning that isn’t shared by a single term or gene This type of network/modular analysis is more in line with the structure of biological data	Tabas-Madrid et al. (2012)

The bold values are the names of software/tools.

### Single-Cell Genomics “Cancer Research/Pan-Cancer Biomarkers”

Single-cell sequencing refers to the sequencing of a single-cell genome or transcriptome in order to gather genomic, transcriptomic, or other multi-omics information that can be used to show cell population distinctions and cell evolutionary linkages as in plethora of cancers. Traditional sequencing methods can only obtain an average of many cells, making it impossible to study a small number of cells and resulting in the loss of cellular heterogeneity data (Wen and Tang, 2018).

Single-cell methods have the advantages of detecting variability among individual cells [1, differentiating a small number of cells, and outlining cell maps when compared to classical sequencing technology (Pennisi, 2012).

Multimodal analysis with integration (Multimodal analysis), or the ability to assess various data types simultaneously from the same cell, is a new and exciting future for single-cell genomics. Weighted closest neighbor (WNN) analysis, an unsupervised technique for learning the information content of each modality in each cell and defining cellular state based on a weighted combination of both modalities, is introduced in Seurat v4. Infact, Multimodal analysis, or the simultaneous measurement of many modalities, is an intriguing new Frontier in single-cell genomics that needs novel computational methods to describe biological states based on numerous data sources. Recent research have demonstrated WNN to create a multimodal reference of human PBMC using a CITE-seq dataset with matched transcriptome and 228 surface protein measurements. WNN can be used to analyse multimodal data from several technologies, such as CITE-seq, ASAP-seq, 10X Genomics ATAC + RNA, and SHARE-seq (Ensslin, 2008) (Tables 8, 9).

**TABLE 8:** Different omics levels of gene-function relationship.

S.No	Level of Analysis	Description	Method of Analysis
1	Genome	Complete set of genes of an organism or its organelles	WGS, WES, DNA microarray
2	Transcriptome	Complete set of messenger RNA molecules present in a cell, tissue of organ	RNA-Sequencing Expression microarray Expression microarray Spatially resolved transcriptomics
3	Proteome	Complete set of protein molecules present in a cell, tissue or organ	Peptide/protein microarrays (RPPA) Mass spectrometry Imaging mass cytometry
4	Metabolome	Complete set of metabolites (low-molecular-weight intermediates) in a cell, tissue or organ	Nuclear magnetic resonance spectrometry Mass spectrometry Infrared spectroscopy
5	Methylome	Complete set of methylation sites within a genome	Bisulfite-Sequencing, ChIP-Seq
6	Microbiome	Complete set of genes of all microbes (bacteria, fungi, protozoa and viruses) in a cell, tissue or organ	DNA-Sequencing 16 S rRNA-Sequencing
7	Lipidome	Complete set of all biomolecules defined as lipids	Mass Spectrometry

WGS, Whole-genome Sequencing; WES, Whole-exome sequencing; ChIP, chromatin immunoprecipitation.

**TABLE 9:** Demonstrates various single cell sequencing technologies.

S.No	Tool name	Description	Ref
1	SCI-seq	Construction of single-cell libraries and detection of cell copy number variation	Vitak et al. (2017)
2	LIANTI	Finding the copy number variation and disease-related mutation	Brierley et al. (2002)
3	scCOOL-seq	Uncovering of chromatin status/nucleosome localization, DNA methylation, copy number variation and ploidy	Guo et al. (2017)
4	Microwell-seq	Enhances the detection abundance of single cell sequencing technology	Han et al. (2018)
5	SPLit-seq	Single cell transcriptome sequencing	Rosenberg et al. (2018)
6	Single-Nucleus RNA-Seq + DroNc-Seq	A variety of cells can be accurately analyzed. It may be used in the Human Cell Atlas Project in the future	Habib et al. (2017)

### Deep Learning in Genomics

Although genomics generates large amounts of data, most bioinformatics algorithms use machine learning and, more recently, deep learning to discover patterns, make predictions, and model disease progression or treatment. Deep learning (DL) advances have sparked a surge of interest in biomedical informatics, spawning new bioinformatics and computational biology research areas. In deep learning models, it is anticipated to deliver higher accuracies in specific genomics tasks than current state-of-the-art methods. Given the growing trend of using deep learning architectures in genomics research. Deep learning will accelerate improvements in genomics. Deep learning is a sort of AI technique that is used to process vast and complicated genomic datasets in particular fields, such as clinical genomics (Koumakis, 2020). Various deep learning architectures have been designed till date, among them includes Artificial Neural Networks (ANN), Convolutional Neural Network (CNN) & Recurrent Neural Networks (RNN).

**Artificial Neural Networks (ANN):** The neurons and networks that make up human brains served as inspiration for Artificial Neural Networks (ANN). The ANN is made up of a set of fully linked nodes (neurons) that simulate the stimulus transmission of brain synapses across the neural network, whether they fire or not. These DL architectures can be used for feature selection, classification, dimensionality reduction, or as a submodule of a more complex design like convolutional neural networks (Zurada, 1992).

**The Convolutional Neural Network (CNN)** is a deep neural network architecture that is most typically used to analyse visual images. It was intended as a completely automated image analysis network for classifying handcrafted characters. CNNs are fully connected networks based on the multilayer perceptrons approach, in which each node/neuron in one layer is (fully) connected to all nodes in the following layer (LeCun et al., 1998).

**Recurrent neural networks (RNN)**: The functioning of recurrent neural networks (RNN) is similar to that of normal feedforward neural networks (FNN), in which nodes form a directed graph along a temporal sequence. RNNs can now demonstrate temporal dynamic behavior while also integrating internal memory. Recurrent networks can remember information from previously studied states thanks to their short-term memory, making them ideal for sequential signal processing and prediction models. The ability of RNNs to relate information from a previous activity to the current task is one of their strengths (Williams and Zipser, 1989a). Table 10 enlists various tools of deep learning (AI) in genomics.

**TABLE 10:** Shows list of deep learning techniques in genomics.

S.No	Tools	Prediction	Ref
1	**DeepTarget**	target prediction	Lee, (2016)
2	**DeepMirGene**	miRNA Target	Park, (2016)
3	**Deep Net**	Case control pre-processing step for clustering. Prediction of transcriptomic machinery	(Gupta et al., 20152015; Dombi et al., 2017)
4	**D-GEX**	Gene expression interference	Chen et al. (2016)
5	**Deep Chrome**	Classify Gene Expression	Singh et al. (2016b)
6	**DeepFIGV**	Predictive Quantative epigenetic variation	Hoffman et al. (2019)
7	**Deepathology**	Predict tissue-of-origin, normal or disease state and cancer type	Azarkhalili et al. (2019)
8	**DeepCpG**	predicts missing methylation states and detects sequence motifs	Angermueller et al. (2017)
9	**DanQ**	predicting the function of DNA directly from sequence alone	Quang and Xie, (2016)
10	**FBGAN**	optimize the synthetic gene sequences	Gupta and Zou, (2019)

The bold values are the names of software/tools.

## Conclusion and Future Perspectives

The introduction of massively parallel sequencing has changed genetics and genomics research forever because of its widespread adoption and numerous applications, massively parallel sequencing is projected to play a vital role in the medical industry in the next years. It is worth noting that NGS as a research tool faces major challenges in terms of manufacturing, data management and downstream analysis.

➢ Thus, in the past decade, rapid advancements in high-throughput intervention, backed by lower costs, have opened up new pathways for interrogating a biological system at several regulatory levels, while also providing us with an unprecedented picture. Integrating more genomic/proteome/transcriptome/metabolome/epigenome data with relevant information obtained at other levels, such as genomes, transcriptomes, epigenomics and metabolomics is still a difficulty.
➢ Nonetheless, new sequencing technologies addressing genomic, proteome, transcriptome, metabolome, and epigenome data clearly have tremendous research potential; their capabilities in the hands of researchers will surely speed our understanding of genomic, medical science and allied domains.
➢ Advances in data creation and analysis skills, as well as the interpretation of outcomes, have pointed to a bright future. However, rapid advancement in all fields of science has resulted in the introduction of novel analytical methodologies. While we continue to learn more about how the body functions, we should shift our focus from molecular to systemic and analytic techniques, which has the potential to revolutionize our understanding of how complex biological systems are regulated.
➢ Data integration, on the other hand, is not the end. Although, the bioinformatics challenges posed by NGS are significant, a variety of software tools and algorithms have been created to aid data management, short-read alignment, and sequence variant identification. The high throughput of NGS necessitates the use of automated pipelines, which aid in the transition from novel sequencing technology
➢ Thus the scenario emphasizes the necessity for scientists who are experts in a variety of fields, as well as the effectiveness of multidisciplinary research groups, in which the complementarity of varied abilities will allow for significant scientific advancements & contributions. Addressing system-wide biological concerns necessitates the use of integrated biology techniques. Routine integration, on the other hand, will necessitate the maturation and alignment of various post-genome technologies, as well as cross-communication across various scientific communities. The effective integration of all of these technologies will eventually lead to next-generation systems biology, which will provide valuable biological insights and adoption to high-throughput research and publication.
